# Dose-response association between dietary fiber intake and hemorrhoid risk among sedentary professionals: a cross-sectional study

**DOI:** 10.3389/fpubh.2026.1842474

**Published:** 2026-07-14

**Authors:** Jing Gao, Dandan Li, Jieping Liu, Fuyan Liu, Shuang Wu, Yufei Zhao

**Affiliations:** Department of International Coloproctological Care, China-Japan Friendship Hospital, Beijing, China

**Keywords:** dietary fiber, dose-response relationship, hemorrhoids, occupational health, sedentary behavior

## Abstract

**Background:**

While prolonged sedentary behavior is an established contributor to hemorrhoidal disease, the precise quantitative impact of dietary fiber within sedentary occupational cohorts remains to be fully elucidated. This study aimed to characterize the dose-response relationship between fiber consumption and hemorrhoid prevalence among professionals in sedentary roles.

**Methods:**

In this cross-sectional investigation of 421 sedentary professionals, dietary patterns were evaluated via validated instruments, and hemorrhoid status was determined through a combination of clinical assessment and symptom-based screening. We utilized multivariable logistic regression to estimate odds ratios (ORs) and 95% confidence intervals (CIs). Furthermore, restricted cubic spline (RCS) analysis, adjusted for age, BMI, exercise habits, and sedentary duration, was employed to model the dose-response landscape.

**Results:**

The cohort exhibited a hemorrhoid prevalence of 38.95%. Multivariable-adjusted models revealed a significant inverse correlation between total fiber intake and hemorrhoid risk (*P* for trend < 0.001). Compared with the lowest energy-adjusted intake group (< 13.1 g/d), participants in the highest quartile (>18.5 g/d) had lower odds of hemorrhoids (adjusted OR: 0.56; 95% CI: 0.32–0.98). RCS regression identified a non-linear “L-shaped” association (*P* for non-linearity = 0.024). The curve showed visual flattening near 25 g/d, which should be interpreted as a descriptive pattern rather than a statistically validated threshold.

**Conclusion:**

Higher dietary fiber intake was independently associated with lower hemorrhoid prevalence among sedentary workers. Because of the cross-sectional design, these findings should not be interpreted as evidence of causality. Prospective studies are needed to clarify temporality and to determine whether increasing dietary fiber intake can reduce future hemorrhoid occurrence.

## 1 Introduction

Hemorrhoidal disease is a common anorectal disorder characterized by bleeding, pain, prolapse, and anal discomfort (1, 2). Its burden is often underestimated because many patients self-treat or avoid medical consultation. Identifying modifiable factors associated with hemorrhoids is therefore important for prevention and symptom control.

The development of hemorrhoids is multifactorial. Disruption of the supporting tissues of the anal cushions, increased intra-abdominal pressure, straining during defecation, and constipation are recognized contributors (3–5). Dietary fiber may influence this pathway by improving stool bulk and consistency, thereby reducing constipation and defecation-related straining (5).

Sedentary work may further increase anorectal risk. Prolonged sitting can increase local pressure and is often accompanied by lower physical activity and slower bowel transit (6, 7). Although clinical guidelines recommend dietary fiber for hemorrhoidal symptoms (8), limited epidemiological evidence has quantified the dose-response association between fiber intake and hemorrhoid prevalence in occupationally sedentary populations.

Therefore, this cross-sectional study investigated the association between dietary fiber intake and hemorrhoid prevalence among sedentary professionals. We further used restricted cubic spline regression to explore whether the association showed a non-linear dose-response pattern.

## 2 Methods

### 2.1 Study design

This multi-center cross-sectional study was conducted in Beijing from January, 2023—December, 2025 to evaluate the association between daily dietary fiber intake and the prevalence of hemorrhoids among sedentary professionals. The research protocol was approved by the Institutional Review Board (IRB) of China-Japan Friendship Hospital. All procedures were performed in strict accordance with the ethical standards of the Declaration of Helsinki and its later amendments. Prior to enrollment, written informed consent was obtained from all participants after a detailed explanation of the study's objectives, procedures, and the voluntary nature of participation. To ensure data integrity and participant confidentiality, all collected information was anonymized and managed through a secure, encrypted database.

### 2.2 Study population

The study population was recruited through a multi-stage cluster sampling method in Beijing. In the initial stage, two or three central urban districts were randomly selected to provide a diverse representation of the regional workforce. Subsequently, specific employment units characterized by high levels of sedentary work, such as corporate offices, software development parks, and governmental institutions, were randomly chosen within the selected districts. All full-time employees within these units were invited to participate. This sampling approach aimed to minimize selection bias and ensure that the cohort was representative of the target occupational group.

The eligibility of the participants was determined by a rigorous screening process conducted by the research team. Inclusion criteria comprised all of the following: (1) adults aged 18 to 60 years; (2) current employment with a sedentary nature, operationally defined for this study as an average occupational sitting time of 6 hours or more per working day, a common threshold in occupational health research and conceptually guided by the Sedentary Behavior Research Network consensus (9); (3) residency or employment in the target area for a minimum of 1 year; and (4) provision of written informed consent to participate in the study. Exclusion criteria encompassed any of the following: (1) a documented history of inflammatory bowel disease (including Crohn's disease or ulcerative colitis) or colorectal malignancy, which may present with symptoms that can confound the clinical diagnosis of hemorrhoids (10); (2) current pregnancy or the early postpartum period, due to the transiently elevated risk of hemorrhoids during these periods (11); (3) presence of severe cognitive impairment or communication barriers that would prevent the accurate completion of the survey; and (4) a history of major anorectal surgery within the past 12 months.

The minimum required sample size was calculated using the formula for cross sectional prevalence studies: *n* = [Z squared ^*^ *p* ^*^ (1 minus p)] / delta squared. Based on the epidemiological findings of Riss et al., the prevalence of hemorrhoids in an adult screening population was identified as 38.93 percent (12). Using a significance level of 0.05 where Z = 1.96 and an allowable margin of error (delta) of 0.05, the calculation yielded a required sample size of 366 participants. To account for a potential 15 percent rate of incomplete data or withdrawal, the final recruitment target was established at a minimum of 431 participants. This sample size provides sufficient statistical power to detect the association between dietary fiber intake and hemorrhoid risk while adjusting for key confounders such as body mass index.

### 2.3 Data collection and variables

#### 2.3.1 General and lifestyle questionnaire

Information was gathered through standardized face-to-face interviews conducted by a team of trained researchers using a customized baseline questionnaire. The questionnaire has been submitted as supplementary material. The English version of this customized questionnaire has been uploaded as a Supplementary file, and relevant details are cited in the main text. The sociodemographic metrics collected included age, gender, self-reported race/ethnicity, marital status, education level, professional role, and average monthly household income. Regarding lifestyle factors, we documented smoking habits and the frequency of alcohol intake. Physical activity was quantified using the validated Chinese version of the International Physical Activity Questionnaire-Short Form (IPAQ-SF) (13). Based on the calculated total metabolic equivalent of task (MET-min/week), subjects were stratified into low, moderate, or high activity categories. Daily sedentary duration was estimated by recording the cumulative hours spent sitting across both occupational and recreational settings. Anthropometric data, including height and weight, were measured to compute the Body Mass Index (BMI) using the standard formula (weight in kg divided by height in meters squared). Furthermore, a multifaceted medical history assessment was performed, covering family history of hemorrhoids and personal conditions such as hypertension and chronic constipation, to account for potential genetic and pathological confounding.

#### 2.3.2 Dietary assessment

Dietary intake over the preceding 12 months was evaluated using a validated, semi-quantitative food frequency questionnaire (SFFQ) comprising 81 food items, which has been established for use in the Chinese population (14). For each item, participants reported their consumption frequency (ranging from never to more than three times per day) and the average portion size using standardized photographic food atlases for reference. The nutritional composition, including total energy intake, macronutrients (proteins, fats, and carbohydrates), and specific fiber types (soluble and insoluble dietary fiber), was calculated based on the China Food Composition Database (7). To ensure the scientific rigor of the dietary data, daily water intake and bowel habits were recorded. Stool consistency was assessed using the Bristol Stool Scale, a seven point scale used to categorize human stool into seven groups based on its shape and appearance, which serves as a proxy for intestinal transit time (15). These factors are critical as they significantly interact with dietary fiber in the pathogenesis of hemorrhoidal disease (11).

#### 2.3.3 Outcome definition

The primary outcome of the study was the prevalence of hemorrhoids. Participants were primarily identified through the core question: “Have you ever been explicitly diagnosed with hemorrhoids by a professional physician?” This physician-diagnosed status was supplemented by a symptom-based screening protocol to capture potentially undiagnosed symptomatic cases. Participants who reported a history of physician-diagnosed hemorrhoids or those experiencing characteristic symptoms, including recurrent rectal bleeding, anal prolapse, or perianal pain during the past 12 months, were categorized into the hemorrhoid group, while those with neither a prior diagnosis nor relevant symptoms were assigned to the non-hemorrhoid group. This combined definition was based on the clinical presentation emphasized in the American Society of Colon and Rectal Surgeons (ASCRS) guideline, which states that hemorrhoidal disease is typically evaluated through disease-specific history and clinical examination, with attention to bleeding, prolapse, perianal pain, bowel habits, and relevant risk factors (2). Because these symptoms are not fully specific to hemorrhoidal disease, we further conducted a sensitivity analysis restricted to participants with physician-diagnosed hemorrhoids.

To ensure the high quality of the study data, all investigators underwent unified training prior to the initiation of the field survey, focusing on the standardization of investigation procedures, questionnaire terminology, and participant privacy protection. Upon the completion of each questionnaire, investigators performed an on site check for logic and completeness. Any missing information or logical inconsistencies were clarified or corrected by the participants immediately. During the data management phase, a double data entry method was employed to minimize transcription errors. Two independent research assistants entered the data into a secure database, and all discrepancies were resolved by cross referencing the original paper forms. This rigorous quality assurance process is widely recognized as a critical standard for maintaining data accuracy in large scale epidemiological investigations.

### 2.4 Statistical analysis

All statistical analyses were conducted using SPSS version 26.0 and R version 4.2.1. Statistical significance was defined as a two-tailed *P* < 0.05 for all tests. Descriptive statistics were employed to summarize the baseline characteristics of the study participants. Continuous variables were assessed for normality using the Kolmogorov-Smirnov test. Normally distributed data were expressed as Mean ± SD and compared between the hemorrhoid and non-hemorrhoid groups using the independent samples *t-*test. Non-normally distributed data were presented as Median (interquartile range) [M (Q1, Q3)], and group comparisons were performed via the Mann-Whitney U test. Categorical variables were expressed as frequencies and percentages [n (%)], and differences between groups were evaluated using the chi-square test.

To investigate the correlation between daily fiber intake and hemorrhoid risk, absolute dietary fiber intake was first energy-adjusted using the nutrient residual method to mitigate the confounding effect of total energy consumption (16). Subsequently, subjects were stratified into four groups based on these energy-adjusted fiber consumption quartiles (Q1–Q4), with the lowest quartile (Q1) defined as the reference. Odds ratios (ORs) and 95% confidence intervals (CIs) were estimated using multivariable binary logistic regression across three incremental adjustment levels: Model 1 (unadjusted); Model 2 (adjusted for demographic factors including age, sex, and BMI); and Model 3 (fully adjusted for education, smoking, alcohol use, physical activity, sedentary behavior, and total energy intake). Constipation history and Bristol Stool Scale type were not included in the primary model because they may lie on the pathway between dietary fiber intake and hemorrhoid development. To assess linear trends across quartiles, the median value of each quartile was entered into the model as a continuous variable. The potential non-linear association between dietary fiber intake and hemorrhoid risk was evaluated using restricted cubic spline (RCS) regression within the multivariable logistic regression framework, rather than by simple descriptive smoothing. This regression-based method was used to estimate adjusted odds ratios and 95% confidence intervals across the continuous range of dietary fiber intake. In the primary RCS model, four knots were positioned at the 5th, 35th, 65th, and 95th percentiles, a commonly used specification in biomedical regression modeling that balances flexibility in detecting non-linearity with model stability (17). The 10th percentile was used as the reference value. Non-linearity was evaluated using a likelihood ratio test comparing the model with spline terms against the model containing only the linear term. To assess sensitivity to knot specification, additional RCS models were fitted using three knots at the 10th, 50th, and 90th percentiles and five knots at the 5th, 27.5th, 50th, 72.5th, and 95th percentiles.

Furthermore, we performed subgroup analyses stratified by sex, age (< 40 vs. ≥40 years), and BMI (< 24 vs. ≥24 kg/m^2^) to identify potential effect modifiers. Interaction effects were examined by incorporating a cross-product term into the regression models. Sensitivity analysis was executed by excluding participants with extreme daily energy intakes (men: < 800 or >4,200 kcal; women: < 600 or >3,500 kcal). An additional sensitivity model was constructed by further adjusting for water intake, constipation history, and Bristol Stool Scale type to evaluate the influence of bowel habit-related variables.

## 3 Results

### 3.1 Baseline characteristics

**Figure 1:**
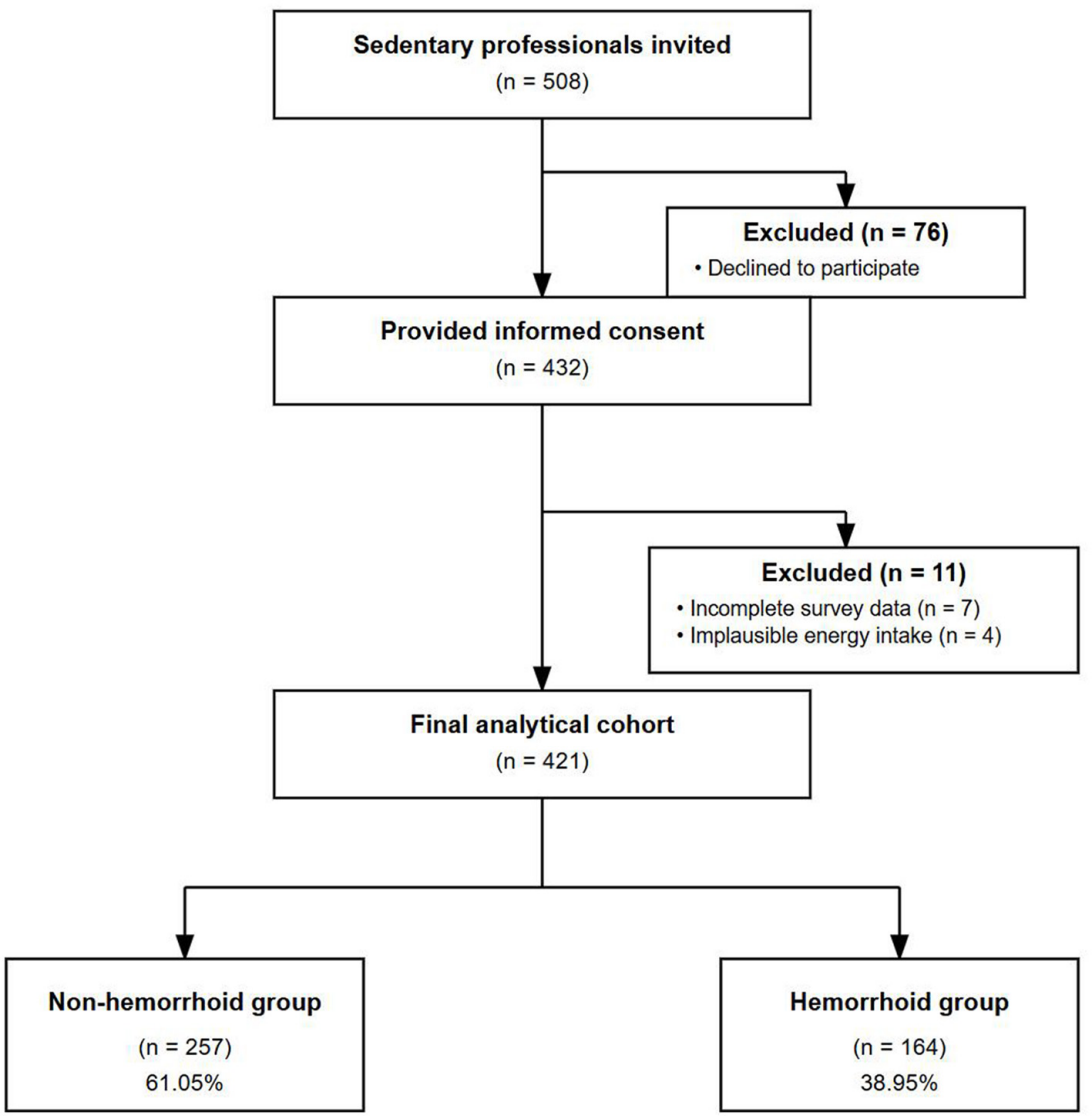
Flowchart of participant recruitment and study exclusion criteria.

The flow of participant recruitment and selection is detailed in Figure 1. A total of 508 sedentary professionals were initially invited, of whom 432 provided consent (85.0% response rate). After excluding 11 individuals due to incomplete data or implausible energy intake, 421 participants were included in the final analysis. Among them, 164 (38.95%) were assigned to the hemorrhoid group and 257 (61.05%) to the non-hemorrhoid group.

**Figure 2:**
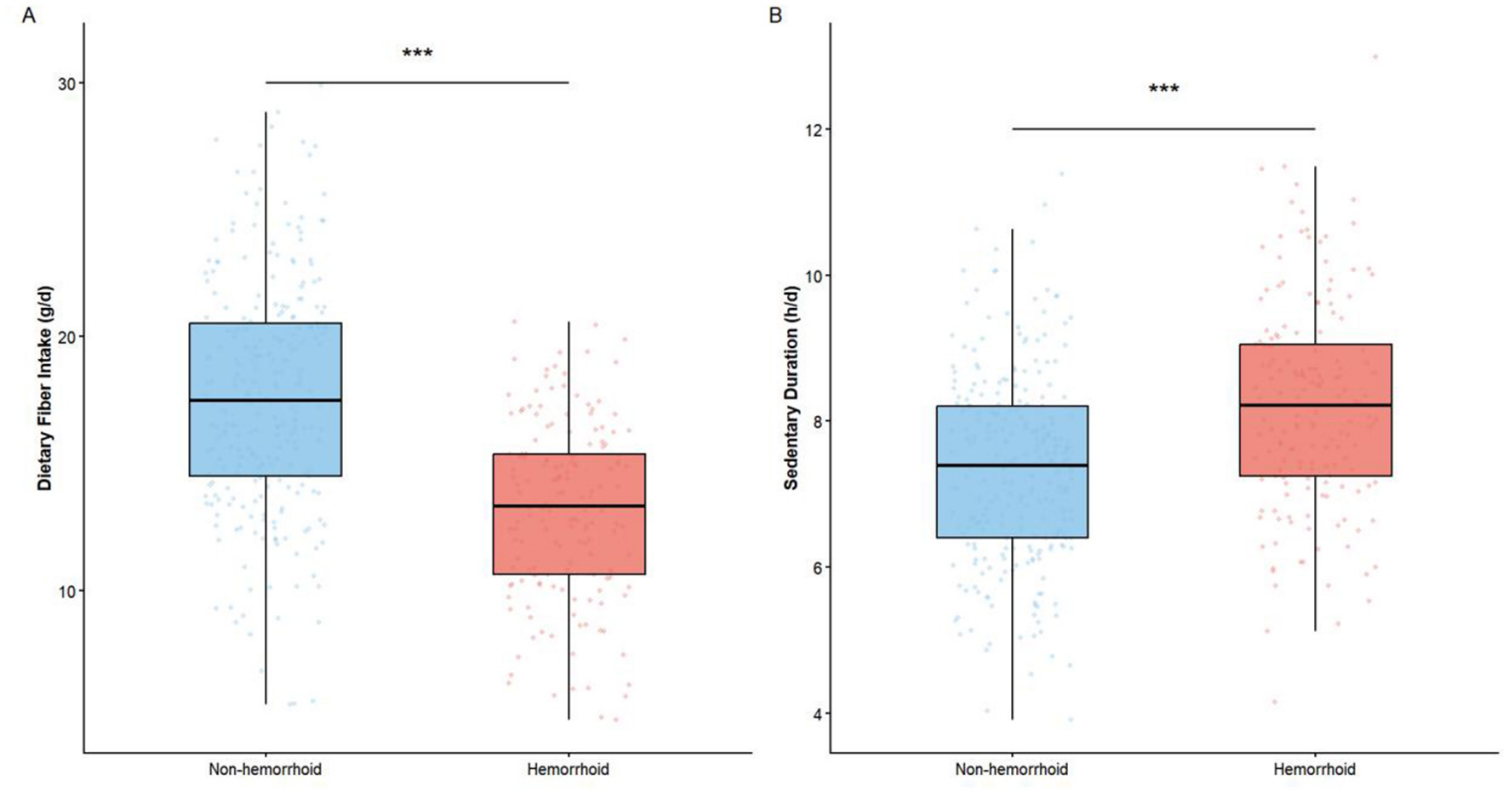
Comparison of daily dietary fiber intake and sedentary duration between the non-hemorrhoid and hemorrhoid groups. **(A)** Daily dietary fiber intake (g/d). **(B)** Daily sedentary duration (h/d). The box plots illustrate the median (center line) and the interquartile range (box limits), with whiskers representing 1.5 times the interquartile range. Individual data points for the 421 participants are displayed as jittered dots to visualize the distribution and density of the data. Blue boxes and points represent the non-hemorrhoid group (*n* = 257), while red boxes and points represent the hemorrhoid group (*n* = 164). Statistical significance was evaluated using the Mann-Whitney U test. ***P < 0.001.

As summarized in Table 1, the hemorrhoid group was characterized by significantly higher mean age and body mass index (*P* < 0.001). Regarding lifestyle and dietary factors, participants with hemorrhoids exhibited longer median daily sedentary durations, lower physical activity levels, and significantly lower median intakes of total, soluble, and insoluble dietary fiber, as well as water (all *P* < 0.001). The key differences in daily dietary fiber intake and sedentary duration are visually highlighted in Figure 2. Furthermore, clinical indicators including chronic constipation, family history of hemorrhoids, and harder stool consistency (Bristol Stool Scale Type 1–2) were significantly more prevalent in the hemorrhoid group (P < 0.001). No significant differences were observed regarding biological sex, marital status, education, occupation, smoking, alcohol use, hypertension history, or total energy and macronutrient intake (all *P* > 0.05).

**Table 1:** Baseline sociodemographic, lifestyle, and clinical characteristics of participants.

Characteristic	Total (*N* = 421)	Non-hemorrhoid (*n* = 257)	Hemorrhoid (*n* = 164)	Statistic	*P*-value
Sociodemographics					
Age (years, mean ± SD)	40.4 ± 9.3	38.2 ± 9.4	43.8 ± 8.3	t = 6.27	<0.001
Sex [female, *n* (%)]	213 (50.6%)	131 (51.0%)	82 (50.0%)	χ^2^ = 0.04	0.844
Race/ethnicity [Asian/Chinese, *n* (%)]	421 (100.0%)	257 (100.0%)	164 (100.0%)	–	–
Marital status [married, *n* (%)]	291 (69.1%)	176 (68.5%)	115 (70.1%)	χ^2^ = 0.13	0.718
Education [college or above, *n* (%)]	374 (88.8%)	229 (89.1%)	145 (88.4%)	χ^2^ = 0.05	0.831
Occupation [technical/IT, *n* (%)]	186 (44.2%)	115 (44.7%)	71 (43.3%)	χ^2^ = 0.08	0.772
Monthly income [>8,000 RMB, *n* (%)]	242 (57.5%)	149 (58.0%)	93 (56.7%)	χ^2^ = 0.07	0.796
Lifestyle and health					
BMI (kg/m2, mean ± SD)	25.0 ± 3.3	24.1 ± 2.9	26.4 ± 3.2	t = 7.64	<0.001
Current smoking [yes, *n* (%)]	74 (17.6%)	44 (17.1%)	30 (18.3%)	χ^2^ = 0.10	0.758
Alcohol consumption [yes, *n* (%)]	104 (24.7%)	62 (24.1%)	42 (25.6%)	χ^2^ = 0.12	0.729
Sedentary time [h/d, median (Q1-Q3)]	7.5 [6.5–9.0]	7.0 [6.5–8.0]	8.0 [7.0–9.5]	U = 14,521	<0.001
Physical activity [low, *n* (%)]	172 (40.9%)	83 (32.3%)	89 (54.3%)	χ^2^ = 20.34	<0.001
Constipation history [yes, *n* (%)]	127 (30.2%)	49 (19.1%)	78 (47.6%)	χ^2^ = 40.52	<0.001
Hypertension [yes, *n* (%)]	88 (20.9%)	51 (19.8%)	37 (22.6%)	χ^2^ = 0.44	0.505
Family history [yes, *n* (%)]	136 (32.3%)	68 (26.5%)	68 (41.5%)	χ^2^ = 10.51	0.001
Dietary and bowel habits					
Energy intake [kcal/d, Med (Q1-Q3)]	2,135 [1,840–2,410]	2,125 [1,835–2,400]	2,150 [1,850–2,425]	U = 20,384	0.527
Carbohydrates [g/d, Median (Q1-Q3)]	282 [245–315]	280 [242–312]	285 [248–318]	U = 19,852	0.321
Proteins [g/d, Median [Q1-Q3)]	77 [65–88]	76 [64–87]	78 [66–89]	U = 20,115	0.508
Fats [g/d, Median (Q1-Q3)]	70 [58–82]	69 [57–81]	71 [59–83]	U = 19,946	0.413
Total fiber [g/d, Median (Q1-Q3)]	15.5 [12.5–19.0]	17.5 [14.0–20.5]	13.0 [10.5–15.5]	U = 11,245	<0.001
Soluble fiber [g/d, Median (Q1-Q3)]	4.8 [3.5–6.2]	5.7 [4.5–6.9]	4.0 [3.2–4.9]	U = 10,852	<0.001
Insoluble fiber [g/d, Median (Q1-Q3)]	10.5 [8.2–12.8]	11.7 [9.5–13.9]	9.0 [7.2–10.8]	U = 12,134	<0.001
Water intake [mL/d, Median (Q1-Q3)]	1,450 [1,100–1,750]	1,550 [1,250–1,800]	1,250 [1,000–1,500]	U = 13,241	<0.001
Bristol Stool Type [Type 1–2, *n* (%)]	114 (27.1%)	43 (16.7%)	71 (43.3%)	χ^2^ = 36.81	<0.001

### 3.2 Association between dietary fiber intake and hemorrhoid risk

As shown in Table 2, dietary fiber intake was inversely associated with hemorrhoid risk. In the unadjusted Model 1, the ORs decreased across increasing fiber intake quartiles (*P* for trend < 0.001). Following adjustment for age, sex, and BMI (Model 2), Q4 showed lower odds of hemorrhoids than Q1.

**Table 2:** Multivariable adjusted odds ratios and 95% confidence intervals for hemorrhoids according to dietary fiber intake quartiles.

Fiber intake quartile	Q1 (reference)	Q2	Q3	Q4	*P* for trend
Energy-adjusted range (g/d)	<13.1	13.1–15.7	15.7–18.5	> 18.5	
Cases/non-cases	65/40	43/62	31/74	25/81	
Model 1 (OR, 95% CI)	1	0.43 (0.25–0.74)	0.26 (0.15–0.45)	0.19 (0.11–0.33)	<0.001
Model 2 (OR, 95% CI)	1	0.48 (0.27–0.85)	0.32 (0.17–0.58)	0.25 (0.13–0.46)	<0.001
Model 3 (OR, 95% CI)	1	0.72 (0.38–1.36)	0.65 (0.34–1.24)	0.56 (0.32–0.98)	<0.001

OR, odds ratio; CI, confidence interval. Dietary fiber intake was energy-adjusted using the nutrient residual method prior to quartile categorization. Model 1: Unadjusted crude model. Model 2: Adjusted for age, sex, and body mass index. Model 3: Adjusted for variables in Model 2 plus educational attainment, smoking status, alcohol consumption, physical activity level, average daily sedentary duration, and total energy intake. P for trend was calculated by assigning the median value of each quartile as a continuous variable in the regression models.

In the fully adjusted Model 3, the ORs for hemorrhoid prevalence were 0.72 (95% CI: 0.38–1.36) for Q2, 0.65 (95% CI: 0.34–1.24) for Q3, and 0.56 (95% CI: 0.32–0.98) for Q4, compared with Q1. Only the highest quartile remained statistically significant in the fully adjusted model. The P for trend was < 0.001. In the supplementary analysis treating energy-adjusted dietary fiber intake as a continuous variable, each 5 g/d increase in fiber intake was associated with a lower prevalence of hemorrhoids (adjusted OR: 0.78, 95% CI: 0.64–0.95; *P* = 0.014; Supplementary Table S1).

### 3.3 Dose-response relationship between dietary fiber and hemorrhoid risk

**Figure 3:**
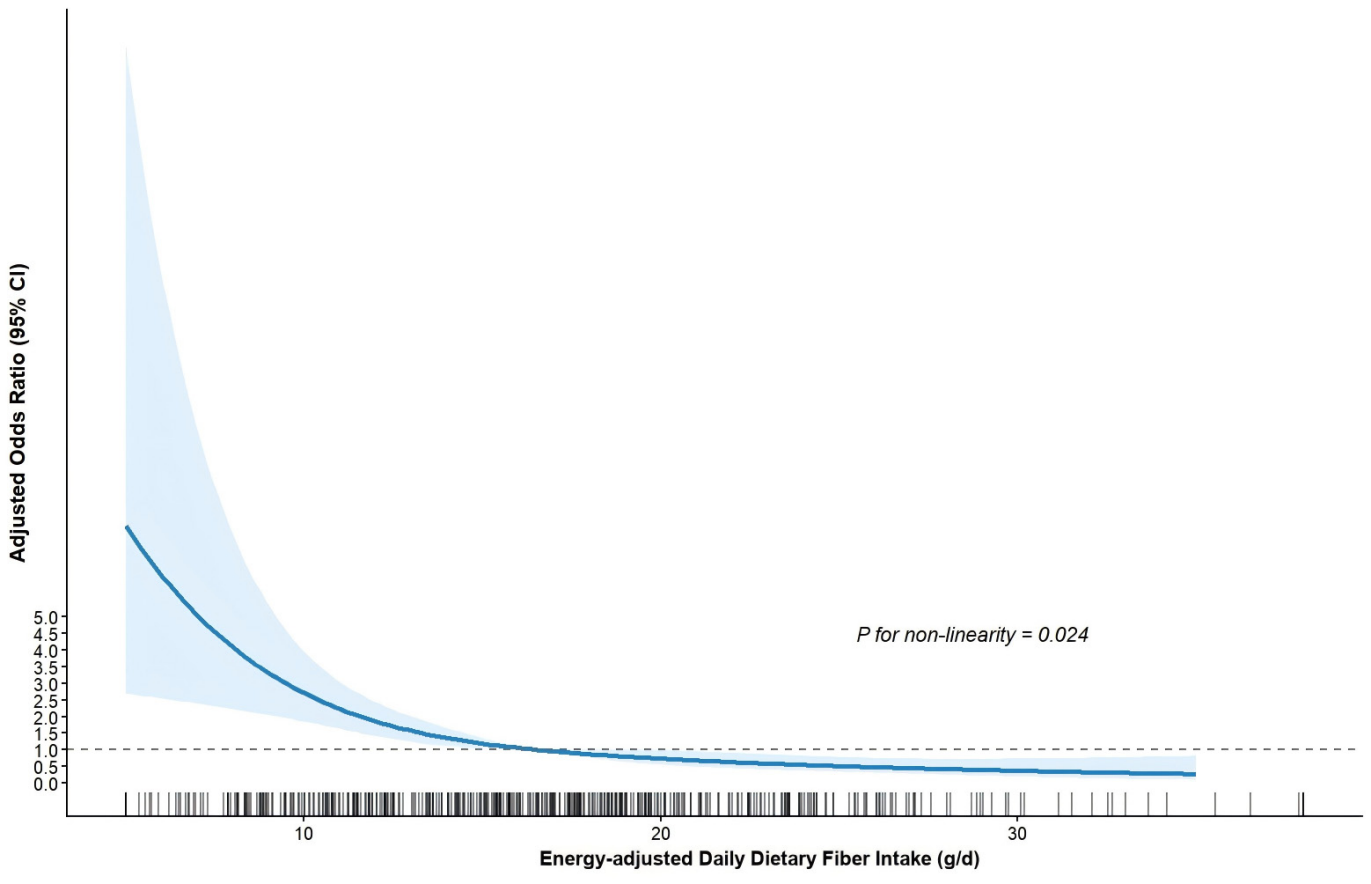
Restricted cubic spline (RCS) regression analysis of the association between energy-adjusted daily dietary fiber intake and hemorrhoid risk (*N* = 421). Dietary fiber intake was adjusted for total energy intake using the nutrient residual method prior to analysis. The dose-response relationship was estimated using RCS terms within a multivariable logistic regression framework, with four knots positioned at the 5th, 35th, 65th, and 95th percentiles of dietary fiber intake. The solid blue line represents the regression-estimated multivariable-adjusted odds ratio (OR), and the light blue shaded area indicates the 95% confidence interval (CI). The horizontal dashed line signifies the reference level (OR = 1.0). The model was fully adjusted for age, biological sex, body mass index, educational attainment, smoking status, alcohol consumption, physical activity level, average daily sedentary duration, and total energy intake. The rug plot along the *X*-axis illustrates the distribution density of daily dietary fiber intake among the participants. *P* for nonlinearity = 0.024. The apparent flattening of the curve near 25 g/d is a visual description of the RCS pattern and was not derived from segmented regression or a formal threshold test.

The dose-response relationship between daily dietary fiber intake and hemorrhoid risk was further characterized using a restricted cubic spline regression model embedded in the fully adjusted multivariable logistic regression framework of Model 3. As illustrated in Figure 3, the RCS regression analysis indicated a significant non-linear association (*P* for non-linearity = 0.024). In sensitivity analyses using alternative knot specifications, the *P* values for non-linearity were 0.038 for the three-knot model and 0.031 for the five-knot model (Supplementary Table S2).

The RCS curve showed a visually apparent flattening around 25 g/d (energy-adjusted), after which further increases in dietary fiber intake were associated with only limited additional reduction in the estimated odds of hemorrhoids. This flattening should be interpreted as a descriptive feature of the RCS curve rather than as a statistically validated threshold or formally tested inflection point.

### 3.4 Subgroup analysis

To assess the consistency of the association between dietary fiber intake and hemorrhoid risk, subgroup analyses were conducted across sex, age, and BMI strata (Table 3). After re-checking the subgroup estimates, the odds ratios, 95% confidence intervals, and *P* values were corrected to ensure internal statistical consistency. The fully adjusted models showed no significant interactions between dietary fiber intake and sex (*P* for interaction = 0.642), age (*P* for interaction = 0.521), or BMI (*P* for interaction = 0.128), indicating that the association did not statistically differ across these prespecified subgroups. The point estimates were consistently below 1.0 across all strata, suggesting a similar inverse direction of association. However, statistical significance was reached only in participants aged ≥40 years and those with BMI ≥24 kg/m^2^, whereas the confidence intervals crossed 1.0 in the male, female, age < 40 years, and BMI < 24 kg/m^2^ subgroups.

**Table 3:** Subgroup analysis of the association between dietary fiber intake (Q4 vs. Q1) and hemorrhoid risk.

Subgroup	Number (H/NH)	OR (95% CI)	*P* value	*P* for interaction
Sex				
Male	82/126	0.58 (0.30–1.12)	0.105	0.642
Female	82/131	0.54 (0.28–1.04)	0.066	
Age (years)				
<40	66/154	0.61 (0.32–1.16)	0.132	0.521
≥40	98/103	0.52 (0.28–0.96)	0.037	
BMI (kg/m^2^)				
<24	48/132	0.65 (0.34–1.25)	0.195	0.128
≥24	116/125	0.48 (0.26–0.88)	0.018	

H, hemorrhoid; NH, non-hemorrhoid; OR, odds ratio; CI, confidence interval; BMI, body mass index. ORs and 95% CIs were estimated using fully adjusted multivariable logistic regression models comparing the highest quartile of energy-adjusted dietary fiber intake with the lowest quartile. All models were adjusted for the same covariates as Model 3 in the primary analysis, except for the stratification variable itself. *P* values were calculated using two-sided Wald tests and were re-checked for consistency with the corresponding 95% CIs. P for interaction was calculated by adding a multiplicative interaction term between dietary fiber quartile and the stratification variable to the fully adjusted model.

### 3.5 Sensitivity analysis

Several sensitivity analyses were conducted (Table 4). When the 11 participants previously excluded for implausible energy intake were included, the OR for Q4 versus Q1 was 0.57 (95% CI: 0.33–0.99). After further adjustment for hypertension, the OR for Q4 vs. Q1 was 0.55 (95% CI: 0.31–0.97). After additional adjustment for water intake, constipation history, and Bristol Stool Scale type, the OR for Q4 vs. Q1 was 0.62 (95% CI: 0.35–1.10; *P* for trend = 0.018). When participants with a family history of hemorrhoids were excluded, the point estimate of the association remained stable (OR: 0.58), although the 95% confidence interval widened to include the null value (95% CI: 0.33–1.02). In the sensitivity analysis restricted to physician-diagnosed hemorrhoid cases, the direction of association remained consistent but the confidence interval was wider (OR: 0.59, 95% CI: 0.33–1.06; *P* for trend = 0.012).

**Table 4:** Sensitivity analysis for the association between dietary fiber intake and hemorrhoid risk.

Analysis scenario	OR (95% CI) for Q4 vs. Q1	*P* for trend
Primary analysis (*N* = 421)	0.56 (0.32–0.98)	<0.001
Including extreme energy intakes (*N* = 432)	0.57 (0.33–0.99)	<0.001
Additional adjustment for hypertension	0.55 (0.31–0.97)	<0.001
Additional adjustment for water intake, constipation history, and Bristol Stool Scale type	0.62 (0.35–1.10)	0.018
Excluding family history of hemorrhoids	0.58 (0.33–1.02)	<0.001
Restricted to physician-diagnosed hemorrhoid cases	0.59 (0.33–1.06)	0.012

OR, odds ratio; CI, confidence interval. All scenarios used the same adjustment framework as Model 3 unless otherwise specified. Extreme energy intake was defined according to the criteria described in the Statistical Analysis section. In the physician-diagnosed sensitivity analysis, participants classified as hemorrhoid cases solely by symptom-based screening were excluded. The additional bowel habit-related adjustment model included water intake, constipation history, and Bristol Stool Scale type.

## 4 Discussion

This study found an inverse association between dietary fiber intake and hemorrhoid prevalence among sedentary professionals. The main finding was a non-linear inverse association between higher fiber intake and lower hemorrhoid prevalence after multivariable adjustment. Specifically, individuals in the highest quartile of energy-adjusted fiber intake (>18.5 g/d) demonstrated a 44% reduction in risk compared to those with the lowest intake (< 13.1 g/d). The lack of statistical significance in the intermediate quartiles may reflect smaller effect sizes, limited separation in fiber intake between adjacent quartiles, and attenuation after adjustment for demographic and lifestyle covariates. Thus, the quartile-based results suggest a graded inverse pattern, with the clearest association in the highest intake group. RCS regression showed an “L-shaped” pattern, with a stronger inverse association at lower intake levels and visual flattening near 25 g/d.

The two-slope appearance is biologically plausible. At low fiber intake levels, even modest increases in dietary fiber may improve stool bulk, stool consistency, and bowel movement frequency, thereby reducing constipation-related straining, a recognized contributor to hemorrhoidal symptoms (2, 18). Once stool consistency and bowel transit have improved, however, additional fiber intake may provide progressively smaller anorectal benefit, which could explain the visually flattened portion of the curve. This pattern is also consistent with evidence that fiber supplementation improves constipation outcomes, particularly when intake is meaningfully increased, but that the magnitude of benefit varies across fiber type, dose, hydration status, and baseline bowel function (18, 19). Therefore, the apparent two-slope relationship may reflect diminishing marginal benefit after a sufficient intake range is reached. However, because no segmented regression or formal threshold test was performed, the value near 25 g/d should be interpreted only as a descriptive reference point, not as a statistically confirmed threshold.

Because no formal threshold test was performed, this flattening should be interpreted descriptively. Alternative three-knot and five-knot RCS models showed similar non-linearity results. Several subgroup estimates were nonsignificant. Although OR point estimates were below 1.0, stratification reduced subgroup sample sizes and widened confidence intervals. This may explain the nonsignificant estimates among males and participants with BMI < 24 kg/m^2^. Hemorrhoidal disease is multifactorial and may be influenced by bowel habits, hydration, sedentary behavior, physical activity, family history, and body habitus (2, 7, 11). Accordingly, associations may be harder to detect in smaller or lower-risk strata. These subgroup findings should be interpreted as exploratory.

The observed association is consistent with current clinical recommendations and previous evidence. The ASCRS guideline recommends increased dietary fiber as a first-line measure for symptomatic hemorrhoids (2). Previous studies also show that fiber supplementation improves bowel regularity and stool consistency in adults with constipation (18, 19), a relevant contributor to hemorrhoidal disease. The hemorrhoid prevalence of 38.95% identified in our sedentary cohort is at the upper end of the globally reported range of 4.4% to 36.4% (20), and notably higher than the 23.9% found in aircraft pilots, another sedentary profession (21). This suggests that office-based sedentary professionals may represent a vulnerable population for hemorrhoidal disease.

The association between dietary fiber and hemorrhoids is biologically plausible. Insoluble fiber increases fecal bulk by absorbing water. This may soften stool, promote intestinal transit, and reduce straining during defecation (22). Constipation and stool consistency may therefore act as intermediate factors rather than simple confounders. Soluble fiber may also support bowel function through gel formation and microbiota fermentation. Its fermentation by colonic bacteria yields short-chain fatty acids (SCFAs), such as butyrate, which nourish colonocytes, maintain gut barrier integrity, and exert anti-inflammatory effects that may further contribute to anorectal health (23). These mechanisms are consistent with clinical evidence that fiber supplementation can improve constipation-related outcomes, although the response varies by fiber type, dose, hydration, and baseline bowel function (18, 19, 24, 25).

From a clinical and public health perspective, these findings are highly relevant. The median dietary fiber intake in our study population was only 15.5 g/d, which is consistent with findings in other populations (26) and falls significantly short of the 25–35 g/d recommended by most dietary guidelines. This “fiber gap” highlights a critical area for intervention. The visually apparent flattening of the RCS curve near 25 g/d may provide a cautious reference point for dietary counseling, but this value should not be interpreted as a definitive clinical cut-off or statistically confirmed threshold. Workplace wellness programs should be encouraged to integrate nutritional education focusing on practical strategies to increase consumption of fiber-rich foods, as this may be a highly effective primary prevention strategy for hemorrhoids and other related gastrointestinal disorders (27, 28).

This study possesses several methodological merits, notably the inclusion of a substantial and well-defined occupational cohort, which ensured sufficient statistical power for our analyses. The use of validated assessment tools for lifestyle factors, combined with sophisticated statistical approaches, such as restricted cubic splines—allowed for a rigorous exploration of the dose-response relationship while meticulously adjusting for key covariates like BMI and sedentary behavior. Nevertheless, certain inherent constraints must be acknowledged. First, the cross-sectional design precludes causal inference. This is particularly important because both dietary intake and symptom-based hemorrhoid screening were assessed over the preceding 12 months. Reverse causality cannot be ruled out, as participants with recent rectal bleeding, prolapse, pain, or other anorectal discomfort may have increased dietary fiber intake for self-management. Because the timing of symptom onset and dietary modification was not recorded, this potential bias could not be quantified. Second, relying on a 12-month self-reported SFFQ is inherently susceptible to measurement error and recall bias. In addition, symptom-based screening may misclassify other anorectal conditions as hemorrhoids. The physician-diagnosed sensitivity analysis showed the same direction of association but wider confidence intervals. Third, the external validity of our findings should be interpreted with caution. Participants were urban Beijing office-based professionals. The findings may not directly apply to rural residents, manual workers, retired populations, or groups with different dietary and lifestyle patterns. Regional differences in fiber sources, hydration, physical activity, healthcare access, and symptom awareness may affect both exposure assessment and hemorrhoid detection. External validation in multicenter and more diverse cohorts is needed. Lastly, residual confounding remains possible. Although water intake, constipation history, and Bristol Stool Scale type were examined in a sensitivity model, straining during defecation and psychological or occupational stress were not collected. These factors should be considered in future prospective studies. Prospective studies are needed to clarify temporality, causality, and the role of specific fiber subtypes.

## 5 Conclusion

This cross-sectional study found an independent, non-linear inverse association between dietary fiber intake and hemorrhoid prevalence among sedentary professionals. The association was most evident at lower intake levels, while the RCS curve showed visual flattening near approximately 25 g/d; however, this pattern should not be interpreted as a statistically validated threshold. Because temporality cannot be established, these findings should be interpreted as associations rather than evidence that higher fiber intake prevents hemorrhoids. Prospective studies are needed to determine whether increasing dietary fiber intake reduces future hemorrhoid occurrence.

## Data Availability

The original contributions presented in the study are included in the article/Supplementary material, further inquiries can be directed to the corresponding author.
