# Supplementary material for: Dose-response association between dietary fiber intake and hemorrhoid risk among sedentary professionals: a cross-sectional study
Source: Front Public Health. 2026 Jul 14;14:1842474. doi: 10.3389/fpubh.2026.1842474 (PMC13407531; doi:10.3389/fpubh.2026.1842474)
Supplement: Supplementary file 1 [file Table_1.docx]

**Supplementary Table S1.** Association between continuous dietary fiber intake and hemorrhoid prevalence

| **Exposure variable** | **Adjusted OR** | **95% CI** | **P value** |
| --- | --- | --- | --- |
| Energy-adjusted dietary fiber intake, per 5 g/d increase | 0.78 | 0.64–0.95 | 0.014 |

Notes: OR, odds ratio; CI, confidence interval. The model was adjusted for age, biological sex, body mass index, educational attainment, smoking status, alcohol consumption, physical activity level, average daily sedentary duration, and total energy intake.

**Supplementary Table S2.** Sensitivity analyses of restricted cubic spline models using alternative knot specifications

| **RCS model specification** | **Knot placement** | **P for non-linearity** | **Curve pattern** |
| --- | --- | --- | --- |
| Primary model | 5th, 35th, 65th, and 95th percentiles | 0.024 | L-shaped pattern with visual flattening near 25 g/d |
| Three-knot model | 10th, 50th, and 90th percentiles | 0.038 | L-shaped pattern with visual flattening near 25 g/d |
| Five-knot model | 5th, 27.5th, 50th, 72.5th, and 95th percentiles | 0.031 | L-shaped pattern with visual flattening near 25 g/d |

Notes: RCS, restricted cubic spline. All models were fitted within the fully adjusted multivariable logistic regression framework, with adjustment for age, biological sex, body mass index, educational attainment, smoking status, alcohol consumption, physical activity level, average daily sedentary duration, and total energy intake. The visual flattening near 25 g/d was not derived from segmented regression or a formal threshold test.
